# Extraction of Phosphate from Polluted Waters Using Calcium Alginate Beads Doped with Active Carbon Derived from* A. aspera* Plant as Adsorbent

**DOI:** 10.1155/2017/3610878

**Published:** 2017-08-28

**Authors:** Ravulapalli Sujitha, Kunta Ravindhranath

**Affiliations:** Department of Chemistry, K L University, Green Fields, Vaddeswaram, Guntur District 522 502, India

## Abstract

An adsorbent prepared by entrapping active carbon derived from the stems of* Achyranthes aspera* plant in the calcium alginate beads (CABAA) has been investigated for its adsorption nature towards the removal of phosphate by varying various physicochemical parameters. Surface morphological studies are made using FTIR, XRD, FESEM, and EDX. The sorption mechanism is analyzed using Freundlich, Langmuir, Dubinin-Radushkevich, and Temkin adsorption isotherms. The adsorption kinetics is found to follow the pseudo-second-order model. Thermodynamic parameters are analyzed and found that the adsorption is endothermic and nonspontaneous in nature. The maximum amount of phosphate adsorbed onto CABAA is found to be 133.3 mg/g of active carbon and, furthermore, the adsorbent is highly selective. The methodology developed is successfully applied to polluted water samples.

## 1. Introduction

The contamination of natural waters with phosphate is considered to be potential environmental problem and in fact, some countries banned the usage of detergents containing phosphates. The sources of phosphate pollution are agricultural runoffs (due to overutilization of fertilizers), domestic sewage wastes (increasing utilization of phosphate containing detergents), and industrial wastes especially pertaining to detergents, food, drinks, and metallurgy [1–4]. The intensive utilization of phosphates produces large amounts of phosphate containing wastewaters and if proper treatment is not given to remove phosphates before the sewage/wastewater is discharged into the environment, there is an accumulation of phosphates in the nearby water bodies near to the point of discharge.

Phosphate causes eutrophication in water bodies leading to serious environmental problems like abundant growth of aquatic plants and varieties of algae and some of which are toxic to the aquatic organisms [5], and further, the DO content in the waters decreases resulting in the loss of aquatic life.

Hence, investigations are being made to find new methodologies to remove phosphate effectively from polluted waters. The conventional treatment methods based on chemical precipitation especially including the use of calcium, aluminium, and iron salts [6–8], though effective in the removal, suffer from problems related to disposal of huge amounts of precipitates. The other traditional techniques such as biological treatment using active sludge processes [9–11], ion exchange [12], electrodialysis, and reverse osmosis [13] are costly.

Bioadsorbents derived from plant materials are intensively being investigated in controlling the pollutants in wastewaters in the recent past. Our research group has developed methodologies based on bioadsorbents to control various pollutants such as chromium (VI) [14], aluminium (III) [15], fluoride [16], nitrite [17], phosphate [18, 19], and dyes [20, 21], and further, the mechanism of oxidation ponds is effectively exploited in removing the toxic ions by cultivating some specific biomasses right in the pond itself [22].

Investigations have been made for the phosphate removal adopting unconventional methods based on red mud [23], La (III) zeolite [24], calcite [25], and metal-loaded orange waste [26]. Agyei et al. [27] used related blends of Portland cement as adsorbent for phosphate removal.

Graphene-lanthanum composites [28], lanthanum-modified bentonite [29], and magnetic diatomite and illite clay [30] were investigated for the removal of phosphate. Altundoğan and Tümen [31] investigated the thermally activated bauxite for the control of phosphate, while Li et al. [32] probed the adsorption nature of acid activated fly ash and red mud towards the phosphate.

Zahid et al. used calcium alginate beads doped with calcium carbonate for the removal of phosphate ions [33]. Han et al. entrapped the hydroxides of Mg and Al in the beads of calcium alginate and studied the extraction of phosphate [34]. Eberhardt and Min studied the nature of biosorbent-polymer treated wood particles towards the adsorption of phosphate [35].

In the present work, active carbon prepared from the stems of* Achyranthes aspera* plant has been entrapped in calcium alginate beads and thus immobilized active carbon in the beads is investigated for its adsorption nature towards the phosphate ions at various conditions.

## 2. Materials and Methods

### 2.1. Plant Description

**Figure 1:**
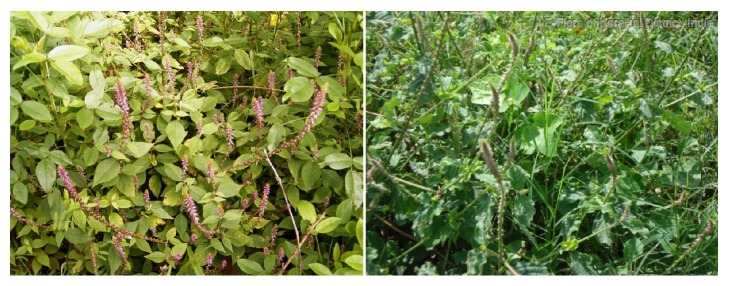
*Achyranthes aspera* plant.

*Achyranthes aspera* (Figure 1), species of Amaranthaceae family, is a common weed and is found in many areas in the southern parts of India. It grows to a height of 15 cm and its parts possesses many therapeutic values especially skin ailments and are diuretic and astringent.

### 2.2. *Achyranthes aspera* Active Carbon Preparation

The stems of* Achyranthes aspera* were cut into fragments, washed with distilled water and sun dried for 3 days. Then the pieces were carbonized using muffle furnace at 250°C for 2 hrs. Thus, obtained carbon was washed with distilled water repeatedly and then filtered and dried at 110°C. Then the substance was grinded and sieved by 75 *μ*m ASTM mesh. The screened carbon was activated by boiling the carbon powder in 1 N HNO_3_ for 2-3 hours on Bunsen burner flame. After treatment with acid, the treated substance was repeatedly washed with distilled water until the washing pH is neutral. Then the substance was dried at 110°C for 5 hrs. The material was preserved in an air tight container for further use.

### 2.3. Preparation of Adsorbent (CABAA)

**Figure 2:**
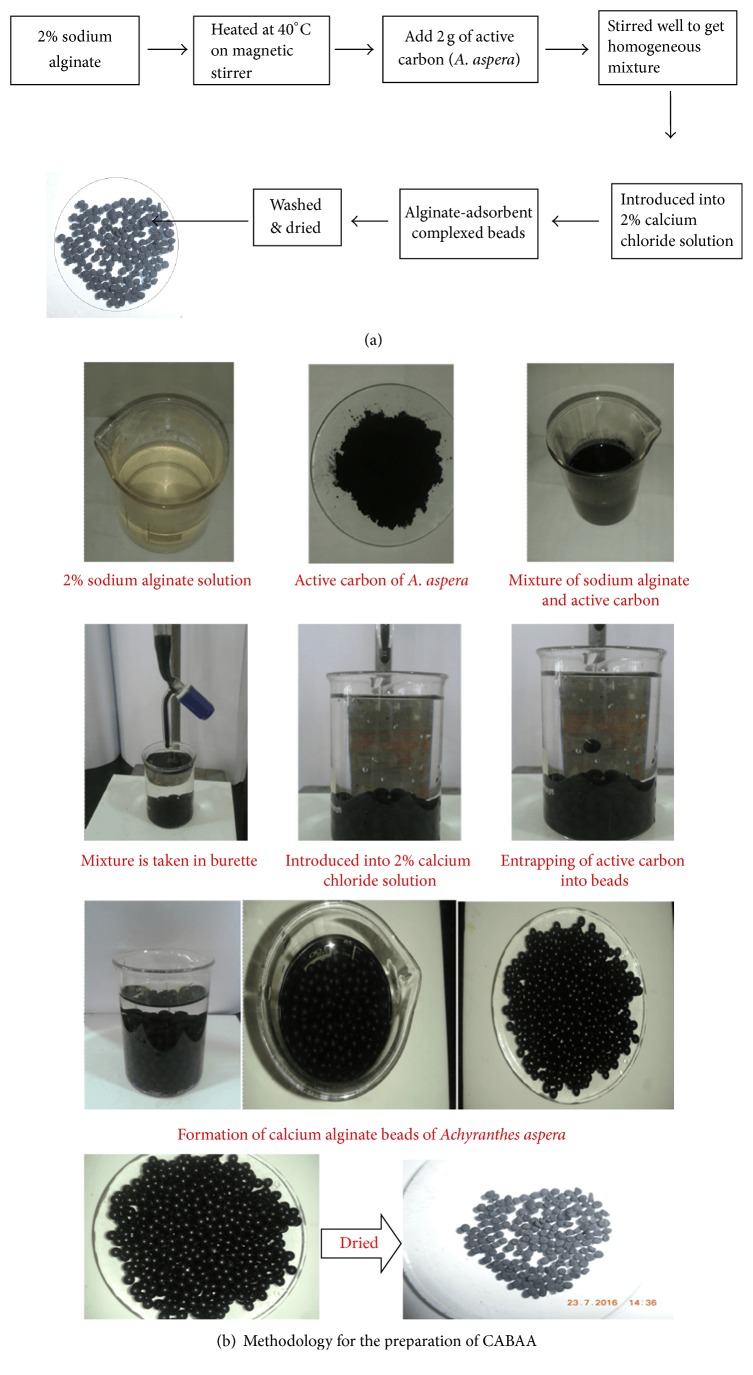


2 g of sodium alginate in 100 ml of distilled water was heated at 40°C; with stirring, a gel-like substance was obtained. To this, 2 g of active carbon of* Achyranthes aspera* was added and the material was stirred further to get a homogeneous mixture. This was cooled and the solution was introduced dropwise into 2% calcium chloride solution with the help of a burette. Fine alginate-adsorbent complexed beads were obtained. Excess of calcium present on beads was removed by washing the filtered beads with distilled water and were dried in oven at 70°C for overnight. The dried beads were named as CABAA (Calcium Alginate Beads of* Achyranthes aspera*) and were used in the present investigation as an adsorbent. The schematic diagram of preparation of adsorbent was represented in Figures 2(a) and 2(b).

### 2.4. Reagents and Chemicals

All the chemicals used are of pure analytical grade. Stock solution of 100 ppm of phosphate was prepared and was diluted as required. Sodium molybdate solution was prepared by dissolving 12.5 g of sodium molybdate in 500 ml of 10 N H_2_SO_4_. Hydrazine sulphate solution was prepared by dissolving 1.5 g of hydrazine sulphate in 1 L of distilled water.

### 2.5. Performance Evaluation of CABAA for Phosphate Adsorption from Aqueous Solution

Batch mode of adsorption experiments [36] was carried out to estimate the extraction ability of the CABAA using simulated 20 ppm phosphate solution.

*Method*. Accurately weighted quantities of CABAA were added to 100 ml of phosphate solution of concentration 20 ppm in 250 ml conical flask at definite pH and the flasks were agitated at 120 rpm. After a certain agitation time, the solution was filtered through Whatman number 1 filter paper and analyzed for phosphate spectrophotometrically by “molybdenum blue” method [37].

*Estimation*. To a sample of phosphate solution in 50 ml volumetric flask, 5.0 ml of sodium molybdate and 5.0 ml of hydrazine sulphate solutions were added and the contents were diluted. Then the flask was heated in water bath for 10 minutes and cooled and the solution was made up to the mark with distilled water. The OD for the developed blue colour was measured at *λ*max: 830 nm using Elico UV and Visible Spectrophotometer and from the obtained OD, the phosphate concentration was calculated referring to the standard graph drawn as per Beer-Lambert's law (between known concentrations of phosphate and ODs adopting linear regression). Using the equations, amount adsorbed (*q*_*e*_) = (*C*_0_ − *C*_*e*_)/*m* × *V* and % removal = (*C*_0_ − *C*_*i*_)/*C*_0_ × 100, the adsorption nature of the CABAA was studied. The parameters in these equations *C*_0_, *C*_*e*_, and *C*_*i*_ are the initial, equilibrium, and final concentration of phosphate in mg L^−1^, respectively, *m* is the mass of CABAA in gs, and *V* is the solution volume in L.

The adsorption nature of CABAA was studied by varying pH, equilibration time, sorbent dosage, temperature, and initial concentration of the adsorbate. The observations were presented in Figures 8–12 and Tables 2 and 3.

Further, the effect of fivefold excess of coanions present naturally in waters on the extraction of phosphate was investigated and the observations were depicted in Figure 15.

## 3. Results and Discussions

### 3.1. Characterization of Adsorbent

#### 3.1.1. FESEM

**Figure 3:**
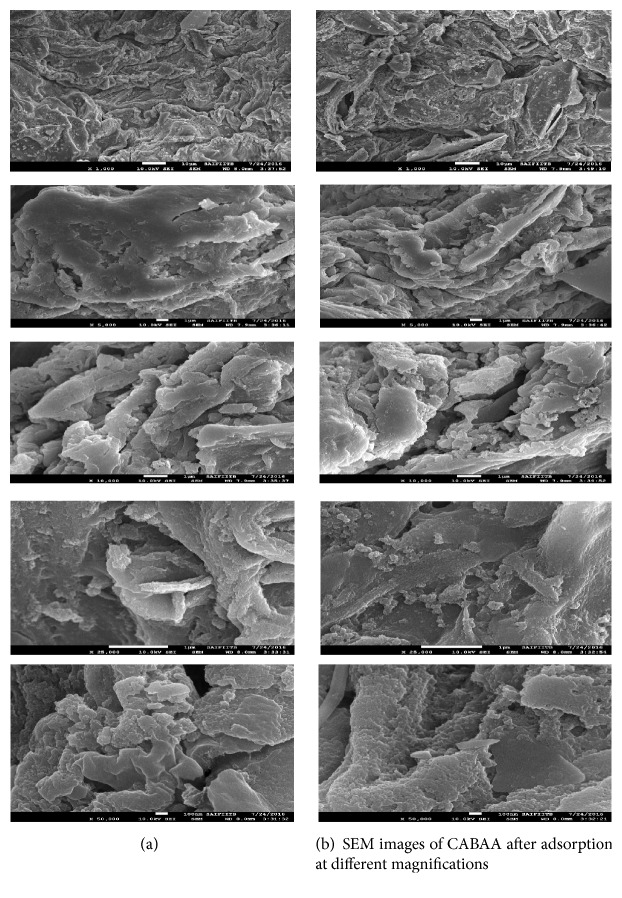


The morphology of CABAA (adsorbent) was observed by FESEM. Monographs of SEM from different areas of CABAA were taken at 10.0 kV with resolutions (1000x to 50,000x) (Figure 3) using JSM-7600F model instrument.

On comparison of the SEM images before and after adsorption of phosphate at high resolutions indicates that the phosphate has been onto the surface of the adsorbent. There is a remarkable difference in the SEM images taken before and after adsorption of the phosphate. It is clearly seen from the SEM images before adsorption of the phosphate that the prepared adsorbent, CABAA, is endowed with pores, fine edges, and many corners. All these constitute the sorption sites for the adsorbate, phosphate. These sites are missing and smooth surfaces resulted in the SEM images after adsorption of the phosphate.

**Figure 4:**
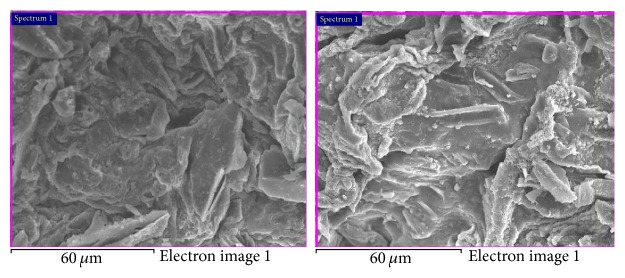
Electronic images of CABAA before and after adsorption.

Furthermore, the presence of phosphate on the surface of CABAA is confirmed by the electronic images of CABAA taken before and after adsorption of phosphate (Figure 4). It is seen from the images that the white composite material is onto the adsorbent along the sorption sites, namely, edges, corners, and pores, and in fact, the porous nature present before adsorption is missing after adsorption in the adsorbent. This clearly concludes that phosphate is on the surface of adsorbent.

#### 3.1.2. EDX

EDS is an analytical technique used for identification and assay (atomic%) of elements present in the samples by virtue of the fact that each element has distinct atomic structure allowing distinct set of peaks on the X-ray spectrum.

**Figure 5:**
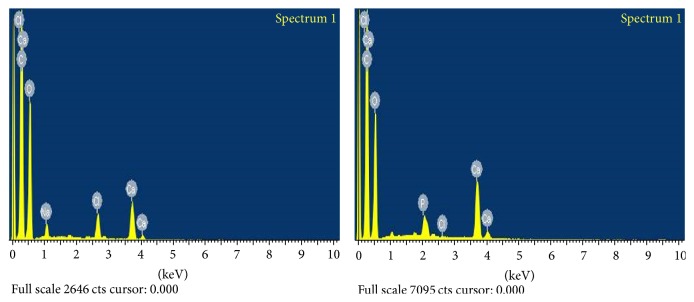
The EDX spectra of CABAA before and adsorption.

In the present work, EDX spectra were taken for CABAA (before and after adsorption of phosphate) and presented in the Figure 5 and Table 1. It is seen from the figure that phosphorus peak appears in the spectrum after adsorption at 2.01 keV as K alpha X-ray signal and this fact indicates the sorption of phosphate onto the surface of CABAA.

#### 3.1.3. XRD

**Figure 6:**
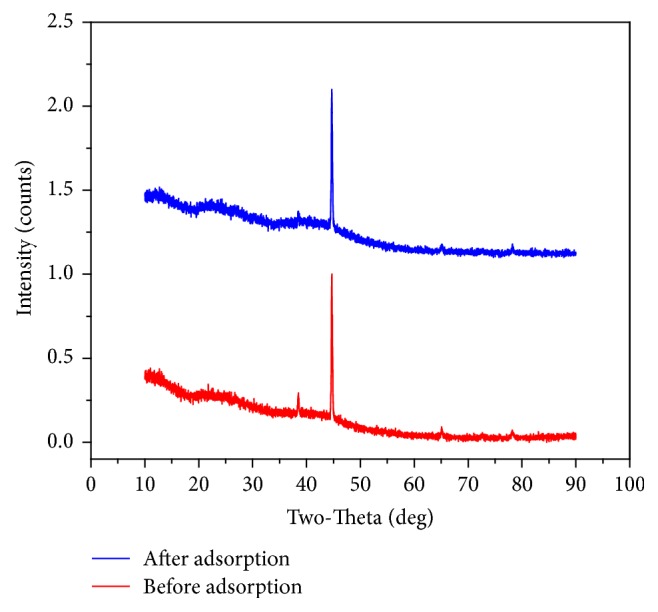
XRD patterns of CABAA before and after adsorption of phosphate.

XRD spectrum is used to understand the crystalline and amorphous nature of the samples. It is observed from Figure 6 that the sharp intensive peaks appear at 2*θ* = 45° in the samples before and after adsorption of phosphate. This indicates that the synthetic method used in the preparation of the adsorbent, CABAA, and the nature of the constituents of the adsorbent do not alter the crystalline phase of adsorbent, CABAA, and even the adsorbate, phosphate, is on the surface.

#### 3.1.4. FTIR

**Figure 7:**
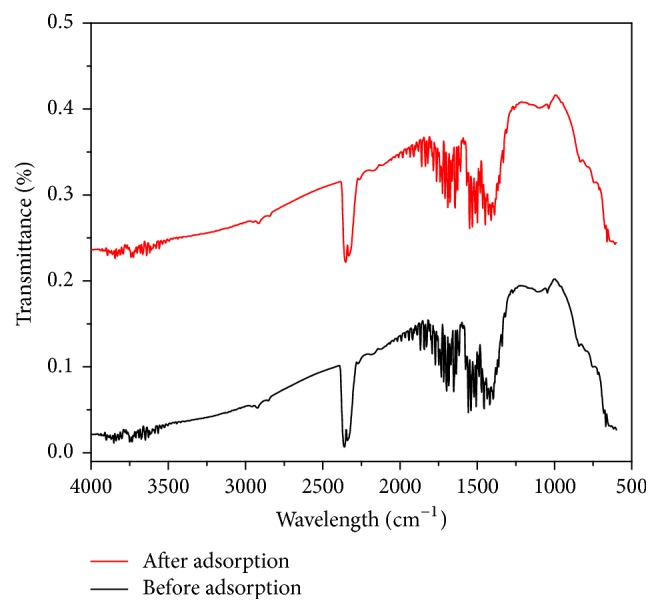
FTIR spectra of CABAA before and after adsorption of phosphate.

Using BRUKER ALFA FTIR spectrometer, the spectral characteristics of the surface of CABAA were examined in the frequency range 4,000 to 500 cm^−1^ to assess the nature of functional groups present. The FTIR spectra were presented in Figure 7.

It is observed that there is no significant difference between FTIR spectra of CABAA taken before and after phosphate adsorption, indicating that the adsorption is ionic in nature and the constituents of the adsorbent selectively interact with the phosphate by ionic interactions.

### 3.2. Effect of Adsorption Parameters on Phosphate Removal

The effect of physicochemical parameters, namely, pH, adsorbent concentration, equilibration time, initial concentration of adsorbate, and temperature on the adsorptive removal of phosphate from simulated wastewater samples, was analyzed adopting batch mode of adsorption methods. Results and discussions are presented hereunder comprehensively.

#### 3.2.1. Effect of pH

**Figure 8:**
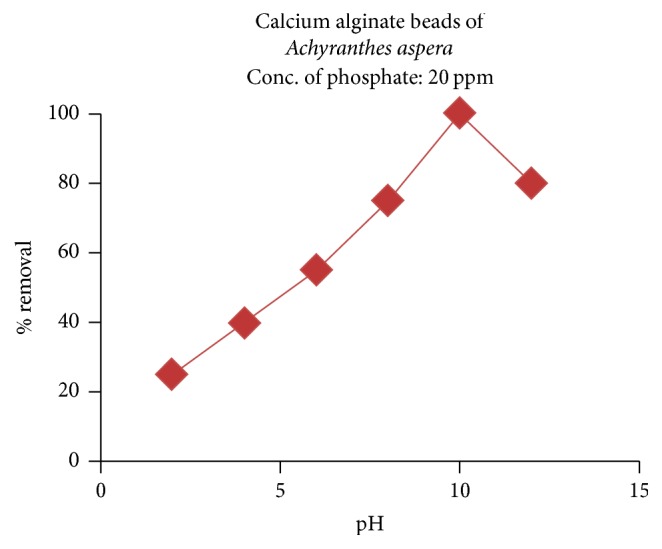
Effect of pH on the adsorption of phosphate ions.

By changing the pH from 2 to 12, the sorption nature of CABAA towards phosphate was investigated while keeping the other conditions of extraction constant, that is, adsorbent dosage: 0.15 g/100 ml, time of equilibration: 20 minutes, initial concentration of phosphate ions: 20 ppm, and temperature: 30°C. The results were depicted in Figure 8. From the figure, it can be inferred that the % removal of phosphate ions is only 25% at pH: 2 and it is sharply increased in between pH 5 and 10 and reached 100% removal at pH: 10, and thereafter, it decreases with further increase in pH.

The observations are as per the nature of speciation of phosphate ions with varying pH of the solution: monovalent, divalent, and trivalent ions present as per the dissociation constants* pK*_1_ = 2.15,* pK*_2_ = 7.20, and* pK*_3_ = 12.33, respectively. In acidic conditions, the predominant species is protonated H_3_PO_4_ and as it is being neutral, it has shown less affinity towards the adsorbent, CABAA, which is doped with active carbons and abridged by positively charged calcium ions possessing residual positive charges at active centres. But as the pH is increased from 5 to 10, the predominance of HPO_4_^2−^, an anion, also increases, which is strongly adsorbed onto the active centres of beads by electrostatic interactions. As the pH is further increased to 12, the hydroxyl ions concentration is high and the ions compete with phosphate ions for the active sites, resulting in low phosphate uptake at pH more than 10.

#### 3.2.2. Effect of Adsorbent Dosage

**Figure 9:**
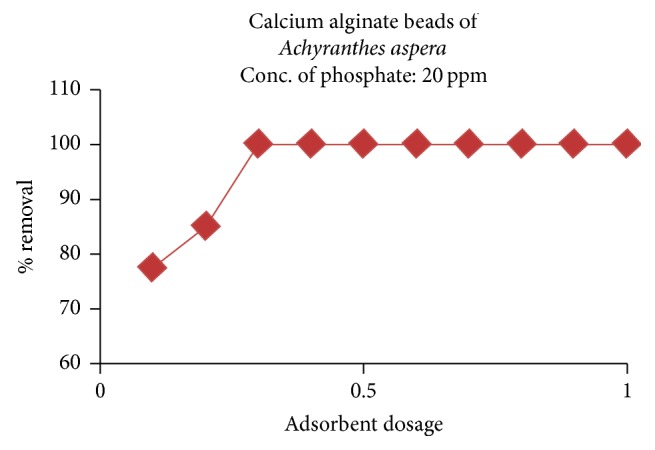
Effect of adsorbent dosage on the adsorption of phosphate.

The effect of adsorbent dosage was studied by varying the sorbent dosage from 0.1 to 0.5 g/100 ml at other optimum extraction conditions: pH: 10; time of equilibration: 20 min; initial concentration of phosphate: 20 ppm; and temperature: 30°C. The results were presented in Figure 9. From the figure, it is inferred that the phosphate removal increases with the increase in concentration of CABAA (adsorbent) but after certain dosage, it remains constant. The increase of adsorption of phosphate until a steady state may be due the increase in sorption sites with increase in sorbent concentration, but at high concentrations of the adsorbent, the mass of the adsorbent blocks some surface active sorption sites and also causes hurdle to the movement of phosphate through the inner pores of the adsorbent.

#### 3.2.3. Effect of Contact Time

**Figure 10:**
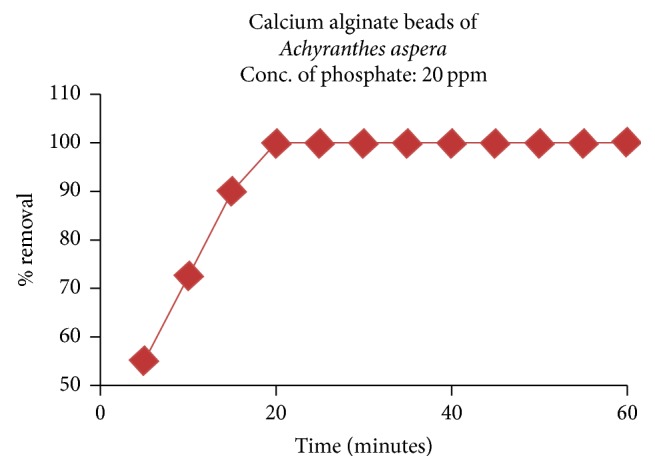
Effect of time of equilibration on the adsorption of phosphate.

% removal was studied as the time was varied from 5 to 30 minutes at pH: 10, adsorbent dosage: 0.15 g/100 ml, initial phosphate concentration: 20 ppm, and temperature: 30°C, and the observations were depicted in Figure 10. It can be seen that the removal of the phosphate increases with increase in agitation time. By 10 minutes itself, 72.5% phosphate ions are removed and then onwards, the removal is slowed down and the 100% removal is attained only after 20 minutes of agitation and after which, there is no significant change in the % removal. The fall of adsorption as the time progresses is attributed to the availability of large number of active sites initially and it is used up as the time progresses.

#### 3.2.4. Effect of Initial Concentration

**Figure 11:**
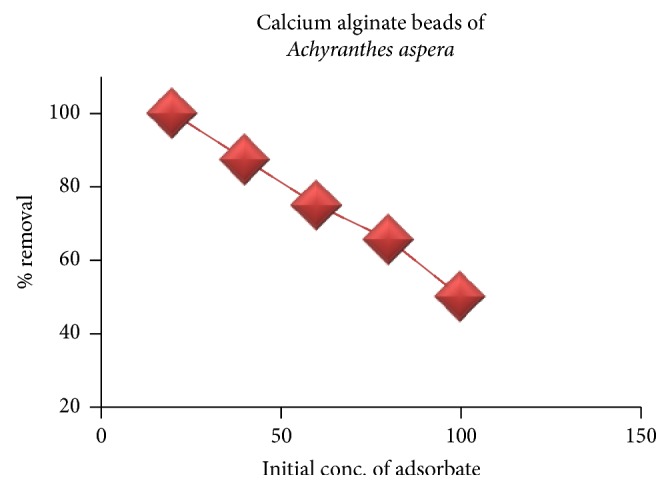
Effect of initial concentration on the adsorption of phosphate.

The effect of initial concentration of adsorbate on the % removal of phosphate ions was studied by varying the initial concentration from 20 ppm to 100 ppm at other optimum conditions of extraction. The results are depicted in Figure 11. From the figure, it is inferred that the % of extraction of phosphate ions decreases from 100% to 50% as the phosphate concentration increases from 20 to 100 ppm. This is because of the reason that, at low concentrations, the active sites of the adsorbent are sufficiently available for the removal of phosphate ions but with increase in the concentrations of the adsorbate, the surface of the active sites is progressively used up resulting in less phosphate uptake.

#### 3.2.5. Effect of Temperature

**Figure 12:**
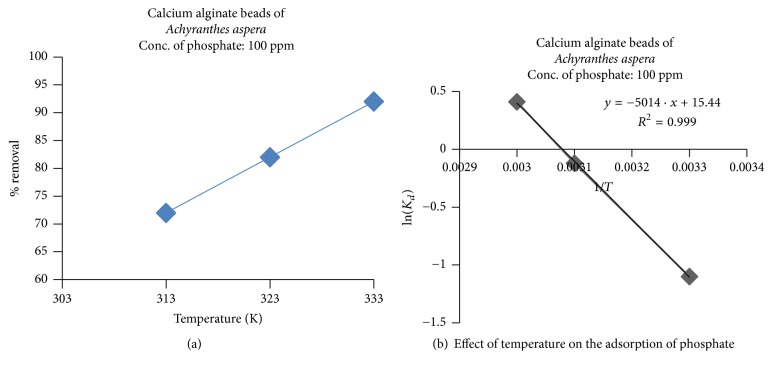


The effect of temperature on the % removal of phosphate ions was studied by taking 100 ppm of simulated phosphate solution and varying the temperatures from 303 to 333 k at other optimum conditions of extraction, that is, pH: 10, contact time: 20 min, and dosage: 0.15 g/100 ml. The results obtained were presented in Figures 12(a) and 12(b) as temperature versus % removal and (1/*T*) versus ln⁡(*K*_*d*_) [38–41].

The thermodynamic parameters, namely, changes in free energy (Δ*G*), enthalpy (Δ*H*), and entropy (Δ*S*), were determined as described in our previous publications [16, 20, 21] and were presented in Table 2.

It is inferred from the figure that, with increase in the temperature, the % removal of phosphate ions is also increased from 72% to 92%. This is due to the fact that, with increasing the temperature, the kinetic energy of the phosphate ions is increased across the internal pores of the adsorbent and, hence, more removal.

It is seen from Table 2 that the Δ*H* values are positive and this indicates the endothermic in nature of adsorption process. Further, the positive values of Δ*S* indicate the increase in disorder and randomness at the interface of adsorbent (CABAA) and adsorbate (phosphate) [42]. Moreover, Δ*G* values are positive indicating the nonspontaneous nature of reaction processes up to 323 K and become spontaneous after 323 K.

### 3.3. Adsorption Isotherms

**Figure 13:**
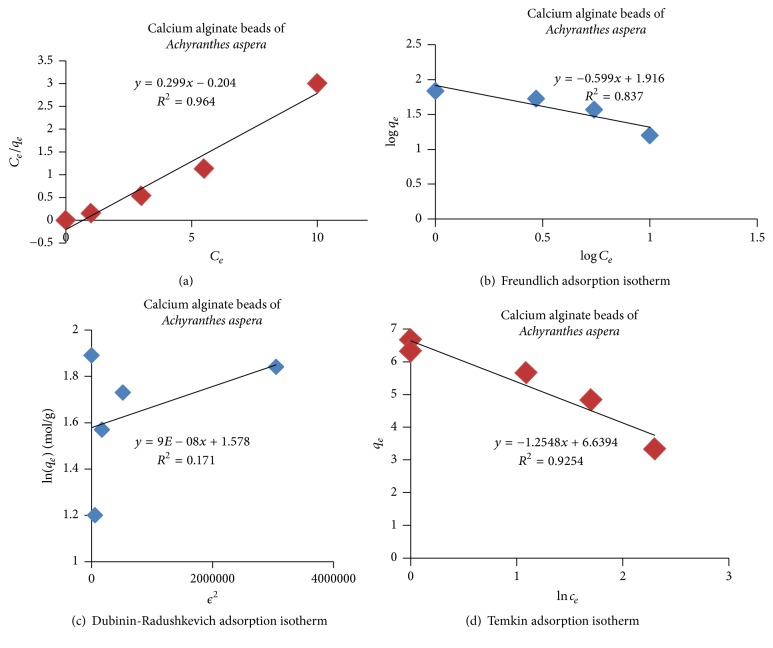


In four well-known models, such as Langmuir [43], Freundlich [44], Temkin [45], and Dubinin-Radushkevich [46], adsorption isotherms were used for analyzing the sorption mechanism of phosphate onto the adsorbent (CABAA) at a constant temperature as described in detail in our previous works [16, 20, 21]. The results were presented in Figures 13(a)–13(d) and Table 3.

The adsorption is unfavourable, linear, favourable, or irreversible if the separation factor, *R*_*L*_ values are >1, =1, between 0 and 1, and zero, respectively [47]. In the present work, the *R*_*L*_ value is 0.033 which is less than unity and hence it indicates the favourability of adsorption process.

Moreover, *R*^2^ (correlation coefficient) value of Langmuir model is 0.964 which is greater than the *R*^2^ values of other models and, hence, the adsorption is well defined by Langmuir model. In summary, the surface of the adsorbent is homogeneous in nature and adsorption process is unilayered.

Further, Temkin constant *B* and mean free energy *E* were calculated as described in our previous works [16, 20, 21]. If *E* value is <8 kJ/mol (reference) and/or *B* < 20 KJ/mol [48], the nature of adsorption is “physisorption” attributed to the weak van der Waal forces present between adsorbate (phosphate) and CABAA (adsorbent). In the present adsorption system, the *E* = 2.35 KJ/mol and *B* = 1.245 J/mole and hence the mechanism of adsorption is only “physisorption” due to electrostatic interactions [49].

### 3.4. Adsorption Kinetics

**Figure 14:**
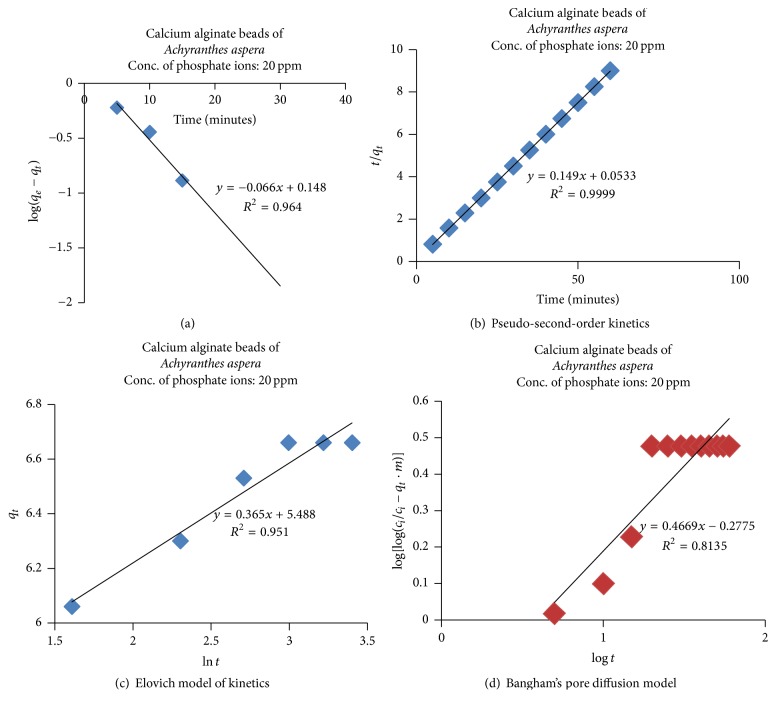


The kinetics of adsorption was analyzed using pseudo-first-order and pseudo-second-order models and Elovich and Bangham's pore diffusion models as described in the literature [50–52] and also in our previous works [16, 20, 21]. The results obtained were depicted in Figures 14(a)–14(d) and in Table 3.

It is observed from Table 3 that, among all the models analyzed, the best fit model is pseudo-second-order model with the correlation coefficient value of *R*^2^ = 0.999. The next reasonable models for describing the adsorption process are pseudo-first-order model (*R*^2^ = 0.964) and Elovich model (*R*^2^ = 0.951) and the least is Bangham's pore diffusion model (*R*^2^ = 0.893). The good correlation coefficient, *R*^2^ = 0.951 for Elovich equation, indicates the diffusion nature and rate limiting factor of phosphate ion onto the surface of CABAA.

### 3.5. Effect of Interfering Anions

**Figure 15:**
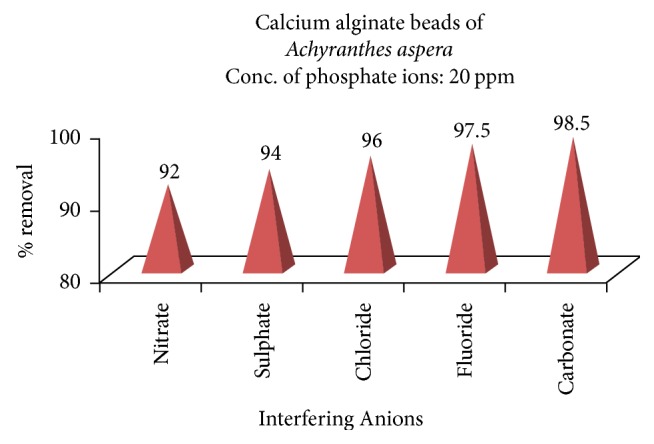
Effect of interfering anions on the adsorption of phosphate.

% removal of the phosphate ions in presence of fivefold excess of the coanions, namely, chlorides, fluorides, sulphate, carbonates, and nitrates, which are generally present in natural waters, were investigated and the observations were shown in Figure 15. It is observed from the figure that the % extractability of phosphate ions was marginally affected by the anions like chlorides, fluorides, and carbonates, while sulphate and nitrates have affected to some extent the % removal. The % of extraction is found to be 92.0% in the presence of nitrate ions and 94.0% in the presence of sulphate.

### 3.6. Applications

The method developed in the present work was applied to real samples collected from polluted water bodies. The phosphate content in them was estimated. Then the samples were subjected to the extraction at the optimum conditions and the results obtained were presented in Table 4.

It is inferred from Table 4 that the present methodology is successful in removing the phosphate to an extent of not less than 90.0% from polluted waters.

### 3.7. Comparative Study of Phosphate Uptake Capacity of CABBA with the Previous Works

Comparative performance of various adsorbents available in literature with the present developed adsorbent CABAA, for the removal of phosphate from polluted waters, was presented in Table 5. It is revealed from Table that the present method is highly successful in removing phosphate to an extent of 133.3 mg/g from wastewater. The method developed is highly selective as is reflected from the interference studies and the adsorbent, CABAA, possesses high adsorption ability towards phosphate when compared with the hitherto reported adsorbents in the literature.

## 4. Conclusions

Active carbon derived from the stems of* Achyranthes aspera* plant has been entrapped in calcium alginate beads and is used as adsorbent (CABAA) for the removal of phosphate from polluted waters. The sorption nature has been investigated by varying the physicochemical parameters such as pH, sorbent dosage, temperature, agitation time, and initial concentration of the phosphate and conditions have been optimized for the maximum removal. It is found that the complete removal of phosphate is observed at pH: 10, initial phosphate concentration: 20 ppm, sorbent dosage: 1.5 g of active carbon in CABAA/L, agitation time: 20 minutes, and temperature: 30°C.

Even fivefold excess of coions such as chloride, fluoride, and carbonate has not effected the % removal, while sulphate and nitrates have effect, but in any case, the % removal has not come down below 90.0%.

Surface morphological studies are made using FTIR, XRD, FESEM, and EDX and it is proved that the phosphate is onto the surface of CABAA.

Adsorption process has been analyzed using Freundlich, Langmuir, Temkin, and Dubinin-Radushkevich adsorption isotherms and it is found that the process can be well described by Langmuir isotherms indicating the homogeneous and unilayered nature of the adsorption process. The adsorption kinetics is found to follow the pseudo-second-order model.

The thermodynamics parameters, namely, Δ*H*, Δ*G*, and Δ*S*, are calculated and found that the nature of adsorption process is endothermic and nonspontaneous. Further, the more adsorption of phosphate with increase in temperature is accounted to the increase in the kinetic energy of phosphate ions resulting in the deep penetration of phosphate ions into the internal pores of CABAA.

The developed methodologies have been applied to the samples collected from natural contaminated lakes and found that they are successful.

The maximum amount of phosphate adsorbed onto CABAA is found to be 133.3 mg/g of active carbon and this adsorbent may turn to be effective in removing phosphate from the polluted waters especially in an agricultural country like India.

## Figures and Tables

**Table 1:** Composition of elements in the adsorbent (CABAA).

EDX of CABAA (before)			EDX of CABAA (after)		
Element	Weight%	Atomic%	Element	Weight%	Atomic%
C K	46.65	56.51	C K	42.02	52.25
O K	43.27	39.35	O K	46.55	43.45
Na K	1.31	0.83	P K	0.33	0.16
Cl K	2.81	1.15	Cl K	0.06	0.03
Ca K	5.96	2.17	Ca K	11.03	4.11

Total	100.00		Total	100.00	

**Table 2:** Thermodynamic parameters of adsorption of phosphate ions on CABAA.

Δ*H* (KJ/mole)	Δ*S* (J/mole)	Δ*G* (KJ/mole)				*R* ^2^
41.68	128.36	2.79 (303 K)	1.50 (313 K)	0.22 (323 k)	−1.05 (333 k)	0.999

**Table 3:** Adsorption and kinetic parameters.

S. number	Adsorption isotherms and kinetic models		Slope	Intercept	*R* ^2^
(1)	Langmuir isotherm	*R*_*L*_ = 0.033	0.299	−0.204	0.964
(2)	Freundlich isotherm	*n* = 1.669	−0.599	1.916	0.837
(3)	Dubinin-Radushkevich isotherm	*E* = 2.357 kJ/mole	9*E* − 08	1.578	0.171
(4)	Temkin isotherm	*B* = 1.245 J/mole	−1.254	6.639	0.925
(5)	Pseudo-first-order model		−0.066	0.148	0.964
(6)	Pseudo-second-order model		0.149	0.053	0.999
(7)	Elovich model		0.365	5.488	0.951
(8)	Bangham's pore diffusion model		0.466	−0.277	0.813

**Table 4:** Applications: extraction of phosphate ions from samples collected from polluted waters using method developed in this work.

S. number	Samples collected at different places	*C*_*i*_ (mg/L) (concentration of phosphate in the sample)	*C*_*e*_ (mg/L) (concentration of phosphate remaining after removal)	% of maximum extraction of phosphate
1	Sample 1	21.5	0.5	97.6
2	Sample 2	25.5	1.5	94.1
3	Sample 3	29.5	2.7	90.8

**Table 5:** Comparative study of phosphate uptake capacity of CABAA with the previous works available in the literature.

Adsorbent	Phosphate uptake capacity (mg/g)	Reference
Alginate-calcium carbonate composite beads	0.72 mg/g	Zahid et al. [33]
LDH-alginate beads	1.558 mg/g	Han et al. [34]
Ashes of leaves of *Annona squamosa*	30 mg/g	Divya et al. [18]
Metal-loaded orange waste	13.94 mg*/*g	Biswas et al. [26]
Acid-thermal treated red mud	0.58 mg/g	Huang et al. [23]
CABAA	133.3 mg/g of active carbon in CABAA	In the present work
